# Cervical cancer programme, Kenya, 2011–2020: lessons to guide elimination as a public health problem

**DOI:** 10.3332/ecancer.2022.1442

**Published:** 2022-08-26

**Authors:** Valerian Mwenda, Woki Mburu, Joan-Paula Bor, Mary Nyangasi, Marc Arbyn, Steven Weyers, Philippe Tummers, Marleen Temmerman

**Affiliations:** 1 National Cancer Control Program, Ministry of Health, PO Box 30016-00100, Nairobi, Kenya; 2 Unit of Cancer Epidemiology, Belgian Cancer Centre, Sciensano, Brussels 1050, Belgium; 3 Department of Obstetrics and Gynaecology, Ghent University Hospital, Ghent 9000, Belgium; 4 Cancer Research Institute Ghent (CRIG), Ghent 9000, Belgium; 5 Department of Human Structure and Repair, Ghent University Hospital, Ghent 9000, Belgium; 6 Department of Obstetrics and Gynaecology, Aga Khan University Hospital, PO Box 30270-00100, Nairobi, Kenya

**Keywords:** cervical cancer, elimination, trends, Kenya, screening

## Abstract

**Background:**

Cervical cancer is the leading cause of cancer mortality in Kenya, with an estimated 3,200 deaths in 2020. Kenya has implemented cervical cancer interventions for more than a decade. We describe the evolution of the cervical cancer programme over the last 20 years and assess its performance.

**Methods:**

We searched the Ministry of Health’s archives and website (2000–2021) for screening policy documents and assessed them using seven items: situational analysis, objectives, key result areas, implementation framework, resource considerations, monitoring and evaluation and definition of roles/responsibilities. In addition, a trend analysis was performed targeting screening and disease burden indicators in the period 2011–2020, using data from Kenya Health Information System and the Global Burden of Disease database.

**Findings:**

Policy guidance improved over time, but the implementation of screening was poor. Before 2016, a clear leadership and accountability structure was lacking; improvement occurred after the establishment of the National Cancer Control Program. The main health system gaps included the lack of a trained healthcare workforce and poor data collection. Annual screening coverage varied between <1% and 36% of the target population for the year for HIV-negative women and between <1% and 7% for HIV-positive women, from 2011 to 2020. Test positivity for visual inspection with acetic acid was below 5% for most of the period. Compliance to treatment of precancerous lesions ranged between 22% and 39%. The detection rate of cervical cancer ranged between 0.5% and 1.0%. The burden of invasive cervical cancer did not change significantly: world age-standardised incidence and mortality rates of 26.3–27.4 and 16.6–18.0/100,000 women-years, respectively; disability-adjusted life years of 579–624/100,000 life years.

**Conclusion:**

The Kenyan cervical cancer control programme suffered from inadequate health system strengthening and poor quality implementation. Evidence-based policy implementation and sustained health system strengthening are necessary to move towards cervical cancer elimination as a public health problem.

## Background

In 2020, the World Health Organization (WHO) launched the Global Strategy Towards the Elimination of Cervical Cancer as a Public Health Problem [1]. The strategy has identified key interventions and targets for countries globally by 2030 (also known as the 90:70:90 cascade): 90% of girls fully vaccinated against human papilloma virus (HPV) by 15 years of age; 70% of women screened with a high-precision test at least twice between the age of 30 and 49 years and 90% of women identified with cervical disease to receive treatment and care (both pre-cancerous lesions and invasive disease).

Sub-Saharan Africa (SSA) has the highest burden of cervical cancer in the world. Africa accounted for 21% of total cases and 26% of global deaths from cervical cancer in 2018 [1, 2]. Cervical cancer is the second leading cause of cancer incidence and the leading cause of cancer death in SSA, accounting for approximately 15% of all cancer deaths in women [3]. While cervical cancer incidence has decreased substantially in high-income countries that have introduced mass screening with high coverage, the burden in SSA stays very high [2, 4]. Many SSA countries have not been able to establish and sustain cervical cancer screening programmes at the population level due to financial, logistical and socio-cultural barriers [5]. Additionally, various healthcare system factors including healthcare worker attitudes, lack of privacy and inadequate further evaluation/treatment facilities have been identified as barriers to cervical cancer screening uptake in SSA, alongside individual and community attributes [6–8]. Screening programmes were often poorly organised and generally did not reach the majority of targeted women [9, 10].

Cervical cancer contributes approximately 12% of all cancer cases diagnosed in Kenya, and is the leading cause of all cancer deaths, with over 3,200 deaths in 2020 [11]. The uptake of screening is low (approximately 16% in 2015) [12] and only a quarter of 2,927 sampled health facilities offered screening in 2018 [13] despite the fact that Kenya has been implementing a national screening programme for more than a decade. Understanding the individual, community and health system factors behind inefficiencies in the programme can guide the country towards the attainment of the global elimination targets. We aim to describe the cervical cancer policy in Kenya over the last decade and assess the trends in screening performance and burden of disease and analyse lessons learnt for future programme improvement.

## Methods

### Study design and location

This was a mixed-methods study design, including both qualitative and quantitative components. The qualitative component involved search and analysis of national cervical cancer policy and practice guidelines published during the period 2000–2021; the quantitative one focused on cervical cancer screening and treatment retrospective data review and analysis, with the data sources being the Kenya Health Information System (KHIS) and the Global Burden of Disease (GBD) database. The study focused on the national programme implementation and performance within Kenya.

### Study period

The document search focused on the period 2000–2021 to demonstrate the policy evolution. However, the retrospective cervical cancer programme data analysis focused on the period 2011–2020, when a structured surveillance system for the programme was available.

### Data sources

A thorough search (both electronic and physical) was conducted, targeting all national policy and practice guidance on cervical cancer screening and treatment in Kenya. Physical documents were sourced from the respective ministry of health departments, including reproductive health and cancer control. Programme performance data was obtained from the cervical cancer control programme (2011–2016) as well as the KHIS. Cervical cancer burden data was obtained from the GBD Study 2019 (GBD 2019), whose cervical cancer burden in Kenya was sourced from the Cancer Incidence in Five Continents, Vol. XI [14] and the Nairobi Cancer Registry report 2004 to 2008 [15]. The datasets from GBD used in this publication are available as supplements.

### Cervical cancer programme performance indicators

The trend of selected indicators over the period of focus (2011–2020) was derived and compared (where applicable) with World Health Organization’s defined standards. These indicators include number of women screened (screening volume), attainment of annual screening targets, screening test positivity (and comparison with the set standards), linkage to treatment, invasive cancer detection, proportion of those within the target age category that are screened, cervical cancer disease burden incidence, mortality and disability adjusted life-years (DALYs) lost. We adopted the standards for screening test positivity from the WHO: 5%–10% for visual inspection with acetic acid (VIA), 5%–25% for HPV testing and 1%–5% (high-grade squamous intraepithelial lesion (HSIL)) for Pap smear cytology.

### Target population definitions

Annual screening coverage was defined as the proportion of screened women against the target number for the year. The screening targets are based on the population structure for the country, as well as HIV burden estimates for women between the ages of 25 and 49 years. Screening targets for HIV negative women/unknown status are calculated every 5 years (incorporating population growth), then divided by the screening interval in years. For HIV positive women, the screening interval is annual; therefore, the same screening cohort has to undergo re-screening in the subsequent year, while incorporating modest increase in the denominator, due to incident HIV cases*.* Since the screening interval for HIV negative women is 5 years as per the Kenya National Cancer Screening Guidelines, two annual target populations were calculated for the periods 2011–2015 (900,000) and 2016–2020 (930,000). This was done through calculation of the number of women 25–49 years from extrapolations from the 2009 census (incorporating population growth) and annualising as guided by the WHO [16]. For HIV positive women whose screening interval using visual methods is annual, the number of women 25–49 years of age out of the overall women living with HIV in Kenya was calculated, and then incorporating annual increases due to incident HIV cases.

### Treatment coverage

The treatment coverage was calculated as a proportion of all screen positive women who received either of the two available treatment modalities in the year under focus (cryotherapy and large loop excision of the transformation zone (LLETZ)), but not necessarily single-visit approach. Only VIA positive cases were considered in the denominator, since HPV testing is also triaged by VIA, and follow-up for positive cytology cases follows a different pathway. The target treatment coverage for the entire period was at least 90% of women with positive VIA.

### Measures of disease burden

Disease burden was described in terms of incidence, mortality and DALYs, modelled from GBD 2019. The burden of disease is computed as a sum of the years of life lost and the years lived with disability, producing the DALYs. DALYs is a better measure of disease impact, especially for chronic conditions. Modelled estimates from GBD 2019 were utilised because the cancer registration system in Kenya was not complete and of high quality to provide cervical cancer disease information over the period under focus in this analysis.

### Data analysis and presentation

A thematic descriptive analysis was conducted on the policy and practice documents, using seven topics: situational analysis, goals and objectives, key result areas, implementation framework, resource considerations, monitoring and evaluation and definition of roles for various actors. The main strengths and gaps were identified. Various interventions implemented over the period were categorised using the World Health Organization health system building blocks [17]. Quantitative data were analysed using Epi Info™ 7.0 (US CDC, Atlanta, GA). The qualitative data was thematically tabulated while the quantitative data was summarised in trend series (bar charts and line graphs).

### Ethical considerations

All the documents analysed in this study are publicly available either electronically or physical copies in the Ministry of Health archives. Only publicly available aggregated cervical cancer screening programme data were utilised for the trend analysis. This work was conducted as part of routine monitoring and evaluation processes at the National Cancer Control Program (NCCP), and therefore did not require ethical clearance.

## Findings

### Policy evolution and implementation milestones

A list of major milestones in the cervical cancer policy and programme implementation is shown in Table 1. Cervical cancer control planning in Kenya started in 2002, when the first strategic plan was drafted. Since then, various initiatives have been launched. However, clear structures for a population-based cervical cancer screening and treatment were lacking. The National Reproductive Health Strategy 2009–2015 sought to address reproductive system cancers; however, it lacked a clear implementation and monitoring framework. In 2011, training on VIA and cryotherapy, as well as distribution of cryotherapy equipment was done. However, subsequent evaluations found that most of the equipment became idle after a few years mostly due to trained personnel attrition and lack of supplies.

The first cancer control strategy (2011–2016) had well-laid out implementation, monitoring and evaluation framework. However, due to lack of a dedicated cancer control agency at the Ministry of Health, the implementation was poorly coordinated. The national Cervical Cancer Prevention Strategic Plan (2012–2015) proposed various interventions in the cervical cancer control continuum. Its implementation lacked a proper governance and evaluation structure and the impact was limited.

Two clinical guidelines were formulated in 2012 (National Guidelines for Prevention and Management of Cervical, Breast and Prostate Cancers) and 2013 (National Cancer Treatment Guidelines). Dissemination to healthcare workers was limited and therefore adoption and application of these guidelines in service provision settings was minimal. A District Health Information System (DHIS) was rolled out in Kenya in 2011. Cervical cancer screening and treatment data became available in DHIS from 2015, making it possible for health managers to track the progress and performance of the screening programme using aggregated data at sub-national and national level. In 2016, two key interventions in cancer control in Kenya were undertaken: (1) the formulation of the National Cancer Control Strategy (NCCS) 2017–2022 and (2) the establishment of the NCCP at the Ministry of Health (MoH). Unlike the previous strategies, the NCCS had a clear governance and implementation framework clarifying the roles and responsibilities for all actors including the national and county governments, other relevant state agencies and non-state actors. The monitoring and evaluation framework included an annual review of the implementation progress. The NCCP provided leadership and served as the coordinating agency. One of the key steps was the establishment of the National STOP (Screening, Treatment, Optimising diagnostics and Prevention) Cervical Cancer Technical Working Group (TWG), which brings together various stakeholders and serves as the think-tank to guide implementation. A number of policy and clinical guidance documents have also been formulated within the implementation of the NCCS, including the National Cancer Screening Guidelines 2018, Kenya Cancer Policy 2019–2030, Cancer Specimen Handling Guidelines and Cancer Treatment Protocols.

HPV vaccination was rolled out nationally in October of 2019, initially targeting 10-year-old girls. Although approximately half of the initial cohort received the first dose, the programme was severely affected by the closure of schools due to the COVID-19 pandemic. The National Cancer Screening Guidelines (2018) recommended HPV testing as the screening modality of first choice. However, the availability of this test was very limited and the cost prohibitive. Therefore, during the 2010–2020 period, the vast majority of women screened had VIA. Interventions towards rolling out HPV-based cervical cancer screening included a pilot in 2019/2020, assessing the feasibility of using GeneXpert as a point-of-care test for HPV, integrating it with TB control programme. This point-of-care test HPV DNA assay has been validated by WHO and fulfils requirements for primary cervical cancer screening [18]. A second pilot was feasibility of HPV sample referral from health facilities to the National Oncology Reference Laboratory (NORL) and relaying of results back to health facilities. Findings from these pilots are meant to guide the MoH on the most practical and cost-effective modality of availing HPV testing across the country. None of the policy documents included provisions on invitation for screening for eligible women. In all the policy documents, the target age group for cervical cancer screening in Kenya is 25–49 years, while screening frequency was every 5 years for HIV-negative women and annually for HIV-positive women, using VIA.

The cervical cancer programme implementation in Kenya over the last decade has had some interventions on each of the WHO health system strengthening blocks, as depicted in Table 2. The impact of these investments, however, was limited by various factors, both within and outside the health system.

### Trends in cervical cancer screening and treatment performance, 2011–2020

#### Population screened per year and annual screening coverage

**Figure 1.**
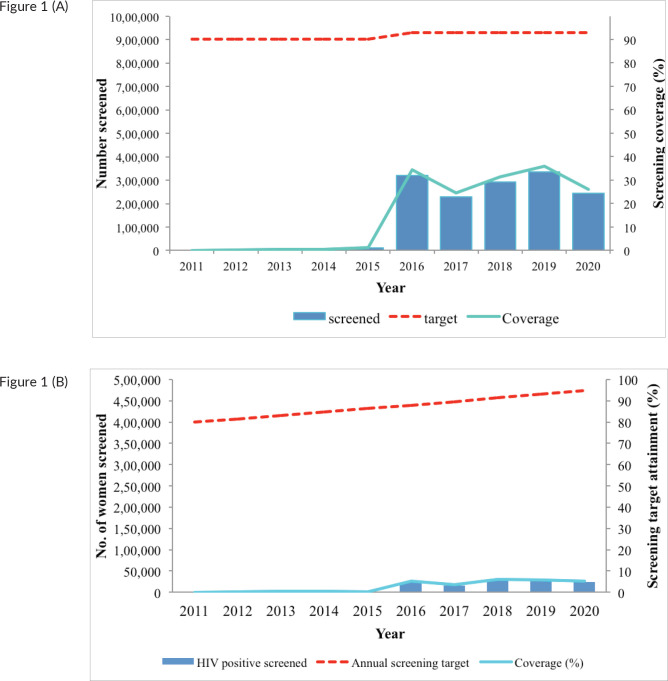
(a): Number of women screened for cervical cancer per year (blue bars), number of women targeted (interrupted red line) and annual screening coverage (green curve) in the HIV-negative population (including also women with unknown HIV-status), Kenya (2011–20). The screening modality used is VIA; with a screening interval of 5 years (Pap smear and HPV testing were used for only a small proportion of women). (b): Number of women screened for cervical cancer per year (blue bars), number of women targeted (interrupted red line) and annual screening coverage (green curve) in the HIV-positive population, Kenya (2011–20). The screening modality used is VIA, with annual screening frequency.

The numbers screened remained very low from 2011 to 2015; however, a sudden increase is noted in 2016, with declines in 2017 and 2020, at the height of the COVID-19 pandemic (Figure 1a and b). Even with the increase in the number of women screened, the annual coverage remained below 40%. The annual coverage among HIV positive women was even lower, below 10% throughout the entire period (Figure 1b).

#### Target population coverage

**Figure 2.**
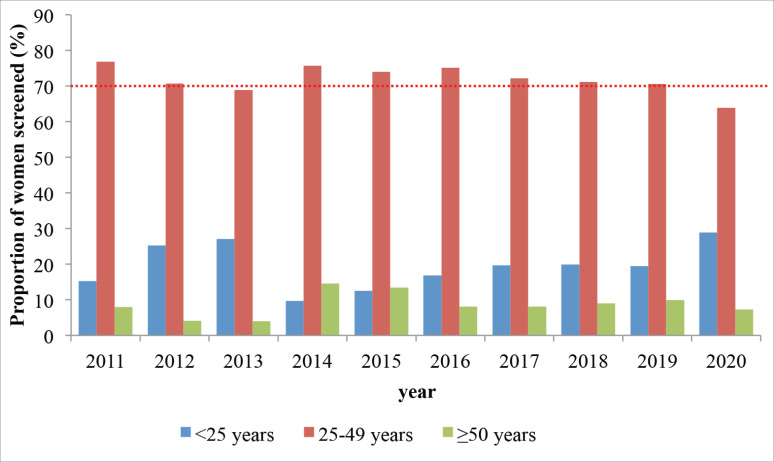
Proportion of screened women who belong to the target age group (red) or is younger (blue) or older (green) than the target age group; Kenya 2011–2020. The dotted red line shows the recommended figure from the WHO (at least 70%).

Across the entire period, 70%–80% of all those screened were between the pre-defined target age category of 25–49 years (Figure 2).

#### Screen test positivity

**Figure 3.**
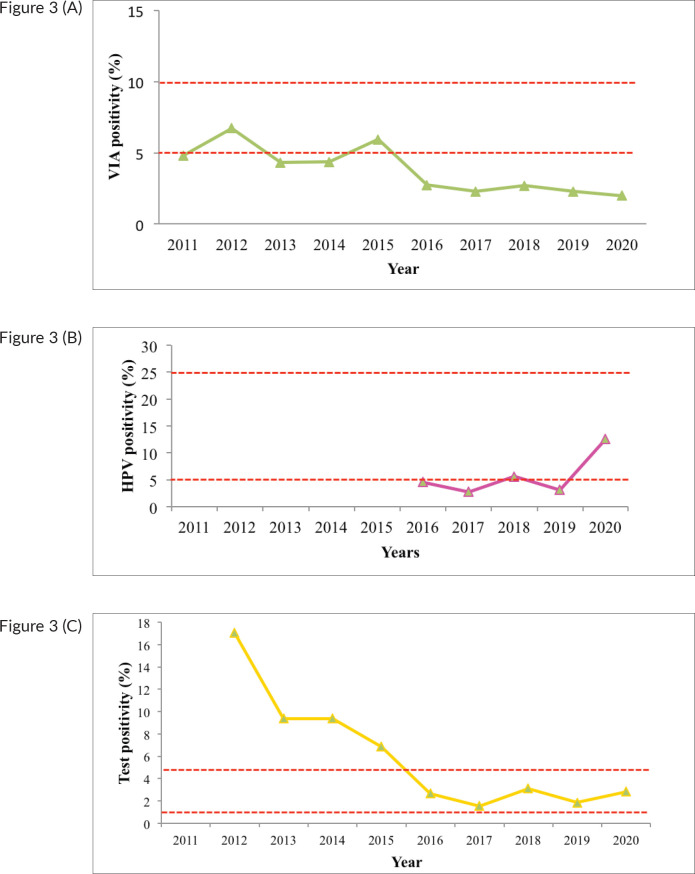
Test positivity, cervical cancer screening programme, Kenya; 2011–2020. (a): VIA test positivity. (b): HPV test positivity; 2011–2020; (c): Cytology positivity; 2011–2020. The red dotted lines represent the expected ranges for test positivity (5%–10% for VIA, 5%–25% for HPV testing and 1%–5% HSIL detection for cytology). HPV testing started in the public healthcare system in 2016; therefore, no data is available before then.

With exception of 2012 and 2015, the VIA positivity remained lower than the expected 5%. VIA positivity has remained within acceptable ranges from 2015 to 2020, after higher-than-expected positivity from 2011 to 2014 (Figure 3). HPV testing, which commenced from 2016, had lower than expected positivity (less than 5%) up to 2019, but an improvement was noted for 2020.

#### Compliance with treatment

**Figure 4.**
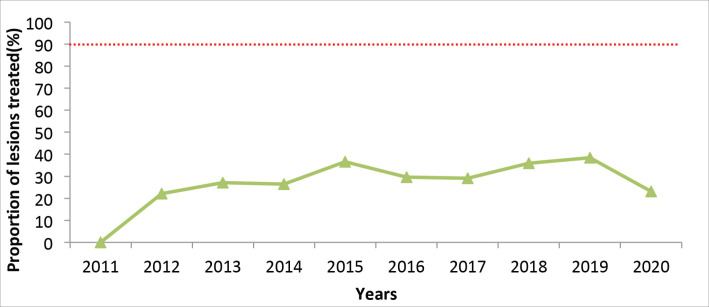
Proportion of cervical pre-cancer lesions detected by VIA who received treatment, Kenya, 2011–2020. The target is 90%, as per the global elimination strategy (dotted red line).

Across the 10-year period, linkage to treatment through cryotherapy or LLETZ for those with positive VIA results has been below 40%, after a steady rise from 2011 to 2015 (Figure 4). This represents those treated within the same year, out of those testing positive. A marked decline is noted in the year 2020 to below 25%.

#### Invasive cervical cancer detection rate

**Figure 5.**
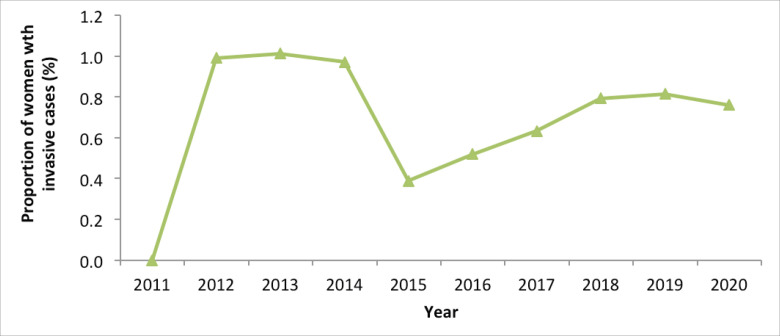
Invasive cancer detection among women presenting at screening centres, Kenya; 2011–2020.

Though also variable, the invasive cancer detection among women presenting at screening centres ranged between 0.5% and 1.0%; the proportion was higher when the numbers of those screened was less than 50,000 (during the earlier years of the implementation of the screening programme) (Figure 5).

### Cervical cancer burden trends, 2010–2019

#### Cervical cancer incidence, 2010–2019

**Figure 6.**
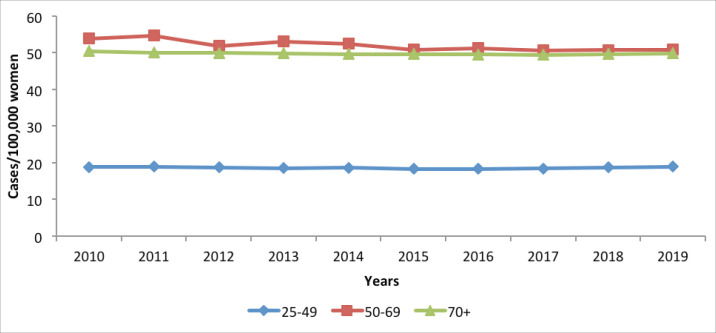
Cervical cancer incidence in Kenya, per 100,000 women, Kenya; 2011–2020.

Cervical cancer incidence is highest among women above 70 years, followed by those in the 50–69 years category (Figure 6). The target age category for screening 925-49 years) had the lowest disease burden in terms of incidence. No appreciable changes are noted in incidence over the 10-year period for the three age categories.

#### Cervical cancer mortality, 2010–2019

**Figure 7.**
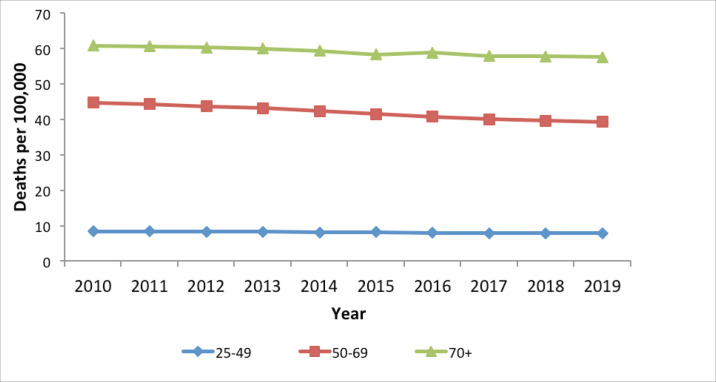
Cervical cancer mortality in Kenya, per 100,000 women, 2010–2019.

Mortality from cervical cancer in Kenya has been declining, albeit slowly, especially for women 50 years above, who have the highest disease burden (Figure 7). Mortality is very low for women 25–49 years of age. A gradual reduction in mortality is noted for women above 50 years of age; however, a rate of above 45/100,000 on average over the period is still very high.

#### Overall disease burden from cervical cancer in Kenya, 2010–2019

**Figure 8.**
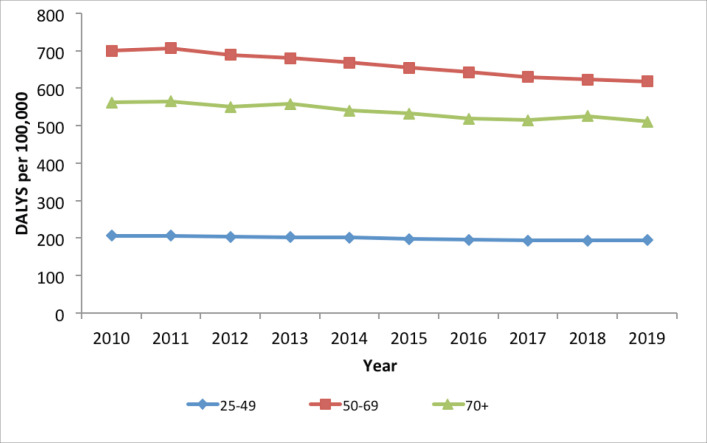
Cervical cancer disease burden in Kenya, in terms of DALYs, 2010–2019.

The burden of disease and mortality from cervical cancer in Kenya is highest in the age category 50–69 years of age (Figure 8). The trends of disease burden in terms of DALYs show a very gradual decrease from 2011 to 2017, especially for the 50–69 years category, but remain relatively unchanged after that. Even though incidence is higher among women of 70 years and above, younger women would live with the disease for longer due to life expectancy (lower mortality), hence higher DALYs.

## Discussion

### Summary of findings

We found that most policy documents lacked clarity in implementation frameworks and monitoring and evaluation frameworks. Most screening and treatment indicators were below set targets over the entire period. These findings can guide Kenya and other low- and middle-income countries in evaluating their cervical cancer policy implementation frameworks, in order to make progress towards elimination.

### Policy formulation and implementation

Quality of policy documents positively evolved over the period, especially in terms of implementation, monitoring and evaluation frameworks. Policy implementation in both reach and depth was affected by decentralisation of healthcare [30]. The separation of roles between national and county levels took time to be implemented and perfected, and this affected the cervical cancer screening and treatment programme. Lack of a dedicated agency for policy guidance and technical support to the counties hampered implementation; an improvement in overall policy dissemination, implementation and evaluation was evident after the NCCP was established. Investments done on the six health systems building blocks were not sustained and built upon in the subsequent years to move the country towards elimination. Clear policy guidance, with an operational and implementation framework is critical for successful cervical cancer control programmes [31]. Systematic evaluation of cervical cancer control programmes is critical in identification of implementation gaps, barriers and opportunities for improvement [32]. We also found no policy framework for individual invitation of women to screening in all the documents studies. A Cochrane review of randomised trials showed that individualised invitations of women eligible for screening increased screening service uptake [33].

### Health system strengthening to support cervical cancer screening

Health system interventions were limited in scope or lacked sustainment to accrue public health impact. Gaps identified include retention of trained personnel, adequate supply of screening and treatment commodities and technologies and a robust health information system supporting the entire invitation-screening-linkage to treatment cascade. These components have been identified as important barriers to cervical cancer screening, on the supply dimension [32]. One consistently improving building block over the decade was leadership and governance; establishment of the NCCP provided a leadership and accountability framework, especially in the last 3 years of the last decade.

### Screening coverage

Screening targets attainment among HIV negative women/unknown HIV status has been consistently below 40%, even after a marked improvement in 2016. This could be due to three main factors. First, there was unavailability of the screening services. A health facility assessment survey conducted in 2018 showed that only 22% of facilities expected to be involved in cervical screening offered the service [12]. Second**,** screening hesitancy, especially among the target age category of 25–49 years, since even with good knowledge, cervical cancer screening uptake has been noted to be hampered by stigma, misconceptions and fear. Third, lack of awareness, not only on the benefits of cervical cancer screening, but also where the screening services are available. This is especially important in rural settings. A systematic review of cervical cancer screening in SSA found the pooled estimate of screening uptake to be approximately 13% [7].

The coverage among HIV positive women was lower than among women with negative/unknown HIV status, in spite that the majority of women living with HIV regularly interact with the healthcare system. While the factors highlighted above could have played a role, another important reason could be lack of fidelity to the screening schedule; once screened a first time, HIV positive women could fall out from annual re-screens, especially if the results of the first screen was negative. Cervical cancer screening uptake among HIV positive women in other SSA countries range from 10% in Ethiopia, 30% in Uganda and 60% in Côte d’Ivoire (though the latter was in an urban setting) [34–36].

### Screening test positivity

The positivity for VIA, the most widely used test, was below 5% for most of the years, especially after 2016, when the screening numbers went up. For most of the years, the positivity was actually below 3%. This low level suggests low sensitivity and should impose urgent corrective action as recommended by WHO target [37]. Test positivity within the recommended ranges is a quality component of the screening programme. A study among female sex workers in Uganda found a VIA positivity of 6% while a pooled estimate of studies in SSA reported a VIA positivity of 17% [2, 38]. A possible explanation for lower VIA positivity in Kenya may be that training and mentorship was not sustained, especially as screening activity increased. With no sustained training and mentorship, the initial cohort of trained primary healthcare workers only passed on skills to colleagues through on-job training, who in turn would do the same to others. Therefore, the skill sets would be eroded with time.

The invasive cancer detection as a proportion of all screened ranged from 0.5% to 1.0 % over the 10-year period; it was higher during the first years of the programme. This is consistent with expected observation whereby advanced cases of the disease are detected more when the screening programme is commencing. An evaluation of the national cervical cancer screening programme in Malawi over a 5-year period found invasive lesions in 4.3% of those screened [39]. The test positivity for HPV remained below 20% for the years when the test was available. This is lower than the Sub-Saharan average of 24.0% as well as Eastern Europe at 21.4% [40]. As a newly-introduced laboratory test, HPV testing could have been affected by weak quality assurance processes; appropriate quality monitoring is necessary to support HPV based screening [41].

### Compliance with recommended treatment

Compliance to treatment remained less than 40%. This includes those who tested positive, postponed treatment but was conducted within the same year. This demonstrates high loss to follow-up up and could also be due to lack of implementation of the Single Visit Approach where women found to have lesions are treated at the same visit. This may also be due to unstructured referrals, since majority of the screening facilities refer positive cases elsewhere for treatment; only 6% of health facilities conduct both cervical cancer screening and treatment. The Malawi program evaluation found that only 40.4% of VIA positive women who were eligible for cryotherapy were actually treated [39]. Replacing cryotherapy with thermal ablation, as recommended by WHO, has the potential to increase the numbers of those treated, as it eliminates the down-time in treatment due to lack of freezing agent.

### Proportion of screened women who were 25–49 years (screened within target age)

Across the period, 70%–80% of women screened were within the target age of 25–49 years of age. This could be explained by sustained awareness creation and health promotion targeting this age group. The WHO recommends a minimum of 70% of all those screened to be within the target age group, where the impact is the largest [37]. Whether a focus on the 50–69 years category, as well as different screening intervals for various age categories would be feasible and efficient in Kenya warrants cost-effectiveness modelling to inform policy revisions.

### Burden of cervical cancer

Cervical cancer incidence and mortality was highest among women 70 years and above, followed by those 50–69 years of age. This can be explained by the long latency period between chronic HPV infection and the development of cervical disease. However, the disease burden in terms of DALYs was highest among the 50–69 years of age category. This could be a result of women in this age category living with the disease for longer after diagnosis, due to longer life expectancy. These findings are consistent with observations made by other studies on the burden of cervical cancer globally [4, 42, 43].

### Strengths and limitation of this study

A particular strength of this study was combination of a policy review and programme performance evaluation. This approach enabled us to evaluate the policy strengths or weaknesses both on the content and the outcomes from screening programme. This study has two major weaknesses. One is the completeness of the quantitative data used, as the health information system has evolved over the decade. Since this would have been more pronounced in the earlier years of the programme, the data was augmented with an evaluation process conducted for the period 2011–2016. The second weakness is although we examined the cervical cancer burden trends (incidence, mortality and DALYs) over the same period, impact on these would be evident after a decade or so. However, the information provided can offer the baseline to measure impact in the current decade. It is also noteworthy that the COVID-19 pandemic in March 2020 caused significant disruptions to the cervical cancer screening programme.

## Conclusion

Cervical cancer screening has not been very successful in Kenya, despite sustained policy and health system strengthening interventions. This was demonstrated by low coverage, test positivity outside expected parameters and ineffective fail-safe mechanisms and linkage to treatment. We recommend action in three areas if Kenya were to progress towards elimination: first, implementation research to give insights on approaches to better implementation of policies; second, innovative approaches to improve participation rate to the recommended 70% for an effective screening programme, invite and link women to screening services; and third, an effective health information system that tracks the screened woman along the screening-treatment-follow-up cascade, with mechanisms to minimise loss to follow-up and encouraging a single visit approach as much as possible. We also recommend periodic systematic monitoring and evaluation of policy implementation in cervical cancer control, and the lessons incorporated in the subsequent policy formulation processes.

## Key points: Review of the cervical cancer policy and implementation journey in Kenya

### Strengths

- The policy, implementation, monitoring and evaluation regarding cervical cancer screening have improved over the last decade.
- Clinical guidance and screening protocols are available, although dissemination has been sub-optimal.
- Some investments have been undertaken in strengthening all the WHO health system building blocks.
- A governance structure for the cervical cancer screening and treatment programme has been established.

### Gaps

- A major gap exists in the implementation of the guidelines and policies, hence the need for implementation research.
- Screening coverage/attainment of screening targets is very low. This includes screening among HIV positive women, despite this being a relatively stable population that interacts with the health system on a regular basis.
- Challenges with quality assurance of the screening programme (test positivity outside the expected ranges), especially for VIA which is the most commonly used method currently. This is important because VIA is the proposed triaging test as the country rolls-out HPV testing nationally.
- Linkage to treatment is low, signalling an in-effective fail-safe mechanism.
- The cervical cancer screening and treatment governance structure was disrupted in the early years of devolution of healthcare tasks to counties.
- Although the health information system has improved over the last decade, it is still deficient of some information, including the following:
  - Single-visit approach rates
  - Linkage to pathology and treatment for those with suspicion for invasive disease
- No systematic evaluation of policy implementation, to guide subsequent polices, therefore opportunities for learning and improvement are lost.

## List of abbreviations

GBD, Global Burden of Disease; KHIS, Kenya Health Information System; HIV, Human immunodeficiency virus; WHO, World Health Organization; HPV, Human papilloma virus; SSA, Sub-Saharan Africa; DALYs, Disease Adjusted Life-years; HSIL, High-grade squamous intraepithelial lesion; LLETZ, Large loop excision of the transformation zone; VIA, Visual inspection with acetic acid; MoH, Ministry of Health; NCCP, National Cancer Control Program; TWG, Technical working group; NORL, National Oncology Reference Laboratory.

## Author contributions

VM conceived the study idea, led data collection and analysis, and wrote the first draft of the manuscript. WM and JB assisted in data retrieval, summarisation and analysis. MN, MA, SW, PT and MT guided data reporting, interpretation and discussion, and gave in-depth review of the manuscript. All the authors read and approved the final version of the manuscript.

## Conflicts of interest

None to be declared.

## Funding

This work was not supported by any specific funding, but was conducted as part of routine evaluation of public health programmes. There are no financial conflicts of interest to disclose.

## Figures and Tables

**Table 1.** Key policy and implementation milestones in cervical cancer control in Kenya.

Title	Year	Type
National Reproductive Health Strategy 2009–2015 [19]	2009	Policy
Provision of 155 cryotherapy equipment distributed in 47 counties	2011	Implementation
NCCS (2011–2016) [20]	2011	Policy
Cancer prevention and Control Act [21]	2012	Policy
National Cervical Cancer Prevention Strategic Plan (2012–2015) [22]	2012	Policy
National Guidelines for Prevention and Management of Cervical, Breast and Prostate Cancers [23]	2012	Clinical guideline
National Cancer Management Guidelines [24]	2013	Clinical guideline
Cervical cancer screening data routinely available in DHIS	2015	Implementation
NCCP established	2016	Implementation
NCCS (2017–2022) [25]	2017	Policy
National STOP Cervical Cancer TWG Sub-committee	2018	Implementation
National Cancer Screening Guidelines [26]	2018	Policy
National Cancer Treatment Protocols [27]	2019	Clinical guideline
HPV vaccination targeting 10-year-old girls	2019	Implementation
Kenya Cancer Policy [28]	2019	Policy
HPV point-of care pilot	2020	Implementation
National Cancer Specimen Handling Guidelines [29]	2020	Clinical guideline
HPV testing phased national scale-up	2021	Implementation
NORL commenced operations	2021	Implementation
HPV sample referral pilot	2021	Implementation

**Table 2.** Status of WHO building blocks of cervical cancer control in Kenya.

WHO health system strengthening block	Interventions undertaken, 2011–2020
Leadership and governance	Initially, the Cervical Cancer Screening Programme was under the Division of Reproductive Health. Decentralisation of health services affected the implementation structure. Establishment of the NCCP within the MoH provided a clear leadership, governance and accountability structure.
Service delivery	Cervical cancer screening in Kenya has traditionally been integrated into reproductive health services, especially family planning. This was practical but limited the coverage of the target population. Integration with HIV treatment followed. Recently, there have been efforts to integrate screening in all health facility service areas, as well as community.
Health system financing	Although no dedicated financing for cervical cancer control has been available in the earlier part of the period under focus, concerted financial investment both from public and donor sources has been witnessed in the last few years, especially during the implementation of the NCCS 2017–2022. After decentralisation, the investment in screening by counties has been minimal, since the concerned units have put more effort in strengthening curative rather than preventive and promotion services.
Health workforce	Over the last decade, several initiatives to increase the capacity of health workers to screen and treat cervical pre-cancerous lesions have been undertaken. Two factors have limited the impact of this investment; disruptions in the health workforce when decentralisation of health services took effect in 2013 and high rate of attrition of trained workforce (especially internal attrition, whereby trained staff are posted to different service points, away from screening centres).
Medical products, vaccines and technologies	Screening commodities like acetic acid have frequent stock-outs at screening facilities, since they are procured differently from medicines. Utilisation of cryotherapy equipment, made available in 2011, has been hampered by a lack of replenishment of the gases regularly (especially at health centres) as well as deficiency of skills. The HPV vaccine was rolled out in October of 2019.
Health information systems	The DHIS was rolled out from 2011, and training and technical support were provided to subnational levels in the following years. Cervical cancer screening information first became available in 2015. The system however lacks critical elements, including linkage to treatment and single-visit approach information.
